# Migratory blackcaps can use their magnetic compass at 5 degrees inclination, but are completely random at 0 degrees inclination

**DOI:** 10.1038/srep33805

**Published:** 2016-09-26

**Authors:** Susanne Schwarze, Friederike Steenken, Nadine Thiele, Dmitry Kobylkov, Nele Lefeldt, David Dreyer, Nils-Lasse Schneider, Henrik Mouritsen

**Affiliations:** 1 AG Neurosensorik/Animal Navigation, Institute of Biological and Environmental Sciences, University of Oldenburg, D-26111 Oldenburg, Germany; 2 Research Center Neurosensory Science, University of Oldenburg, D-26111 Oldenburg, Germany

## Abstract

It is known that night-migratory songbirds use a magnetic compass measuring the magnetic inclination angle, i.e. the angle between the Earth’s surface and the magnetic field lines, but how do such birds orient at the magnetic equator? A previous study reported that birds are completely randomly oriented in a horizontal north-south magnetic field with 0° inclination angle. This seems counter-intuitive, because birds using an inclination compass should be able to separate the north-south axis from the east-west axis, so that bimodal orientation might be expected in a horizontal field. Furthermore, little is known about how shallow inclination angles migratory birds can still use for orientation. In this study, we tested the magnetic compass orientation of night-migratory Eurasian blackcaps (*Sylvia atricapilla*) in magnetic fields with 5° and 0° inclination. At 5° inclination, the birds oriented as well as they did in the normal 67° inclined field in Oldenburg. In contrast, they were completely randomly oriented in the horizontal field, showing no sign of bimodality. Our results indicate that the inclination limit for the magnetic compass of the blackcap is below 5° and that these birds indeed seem completely unable to use their magnetic compass for orientation in a horizontal magnetic field.

It is well known that birds fly long distances between their breeding and overwintering grounds, and, for the last 60 years, many scientists have worked on understanding the fundamentals of this phenomenon. Migratory birds possess separate compass mechanisms based on the sun^1^,^2^,^3^, the stars^4^,^5^,^6^,^7^,^8^, and the magnetic field^2^,^9^,^10^,^11^,^12^. In this paper, we studied the magnetic compass of migratory blackcaps (*Sylvia atricapilla*, Linnaeus 1758).

Currently, there are two major hypotheses suggesting how birds can sense the Earth’s magnetic field. One hypothesis suggests that the ophthalmic branch of the trigeminal nerve is involved in magnetoreception^13^,^14^,^15^,^16^,^17^,^18^,^19^. This hypothesis has often been associated with iron-mineral-based receptors^20^,^21^ but see ref. 22 and 23. The other hypothesis suggests that light-dependent, radical pair processes in both of the birds’ eyes are responsible for magnetoreception^24^,^25^,^26^,^27^,^28^,^29^,^30^,^31^,^32^,^33^,^34^,^35^,^36^,^37^,^38^,^39^. While a magnetite crystal should be able to sense the polarity of the field lines, the radical-pair mechanism would only be able to sense the axis of the field lines and could therefore only act as an inclination compass^9^,^11^,^24^,^31^,^38^.

Meanwhile, it is well documented that the birds’ magnetic compass is an inclination compass^9^,^10^. This means that the compass capabilities of birds are not based on the polarity of the Earth’s magnetic field, like technical compasses built by humans, but on the angle of the Earth’s magnetic field lines in relation to the gravity vector or the Earth’s surface^9^,^10^. The magnetic inclination angle changes from 90° at the magnetic poles to 0° at the magnetic equator. The smaller angle between the magnetic field lines and the Earth’s surface always points towards the magnetic equator, whereas the largest angle between the magnetic field lines and the Earth’s surface always points towards the magnetic pole. Thereby, an inclination compass does not differentiate between north and south, but between poleward and equatorward^9^,^10^,^11^. However, many species have to cross the magnetic equator during migration^40^,^41^,^42^,^43^. Consequently, these birds must be able to switch their orientation from “equatorward” to “poleward” after crossing the magnetic equator. Wiltschko and Wiltschko^44^ and Beason^45^ suggested that experiencing the zero-inclination at the magnetic equator could trigger this reversal. Beason^46^ and Cochran *et al*.^2^ suggested that birds use different cues than the magnetic compass to cross the “blind zone” at the magnetic equator and that they might calibrate their magnetic compass anew from celestial cues after the crossing. But how can trans-equatorial migrants orient at the magnetic equator itself?

Because the inclination angle is 0°, the birds cannot distinguish between “poleward” and “equatorward” at the magnetic equator by using their magnetic inclination compass. Wiltschko and Wiltschko^9^ and Wiltschko^47^ reported that birds using a magnetic inclination compass for orientation are randomly oriented in a magnetic field with 0° inclination. We found this result somewhat surprising, because even though the birds should not be able to discriminate north from south, they should be able to differentiate between the north-south axis and the east-west axis (see Fig. 1).

Consequently, we decided to attempt a double-blinded, independent replication of the previous findings. Our aim was to test the magnetic compass orientation capabilities of night-migratory Eurasian blackcaps in a magnetic field with 0° inclination. We also tested the birds in the natural geomagnetic field of Oldenburg (67°) and in a magnetic field with a very shallow inclination angle (5°). In addition to the double-blinding, other important strengths of the present study were the permanent monitoring and the meticulous control of the magnetic stimulation, which was made possible through the use of computer controlled, three-dimensional Merritt-four-coil systems^11^,^28^,^48^ creating very homogeneous magnetic fields within the chambers.

## Results

In spring 2014, the blackcaps oriented significantly in their appropriate migratory direction when they were tested in a magnetic field pointing towards magnetic north with a magnetic inclination of 5° (5°NMF: group mean orientation = 47° ± 37° [95% confidence interval], *r* [group mean’s vector length] = 0.61, *N* = 11, *p* < 0.05 [Rayleigh-test], Fig. 2a). When the horizontal component of the 5° inclined field was rotated −120°, the birds turned their orientation accordingly (5°CMF: group mean orientation = 294° ± 57°, *r* = 0.57, *p* < 0.05, *N* = 11, Fig. 2c). The Mardia-Watson-Wheeler test showed a significant difference in the orientation direction between 5°NMF and 5 °CMF (*W* = 6.402, *df* = 2, *p* = 0.041). In contrast, the birds were randomly oriented in both the north-pointing and the −120° rotated field when the inclination angle was set to 0° (spring 2014 0°NMF: group mean orientation = 16°, *r* = 0.26, *p* = 0.486 (ns), *N* = 11, Fig. 2b, 0 °CMF: group mean vector = 360°, *r* = 0.36, *p* = 0.214 (ns), *N* = 12, Fig. 2d). However, the distribution in the 0° inclination seemed to be not truly random, but bimodal in the 0°NMF condition (doubled angles: group mean vector = 120°/300°, *r* = 0.65, *p* < 0.01, *N* = 11), whereas there were no signs of bimodal orientation in the 0°CMF condition (group mean vector = 127°/307°, *r* = 0.31, *p* = 0.347 (ns), *N* = 12). The possible bimodal orientation in the 0°NMF condition is not oriented along the expected NE-SW axis. Therefore, we decided to test the birds again in both conditions in the following spring.

In autumn 2014, we tested blackcaps in the 5°NMF and 5 °CMF conditions as well as in a control condition, where the inclination angle corresponded to the natural geomagnetic field of Oldenburg (67° inclination, 67°NMF). The condition with 0° inclination was not used during the autumn season in case it would function as a migration stop or migration direction reversal cue^44^,^45^. The blackcaps were significantly oriented in their appropriate migratory direction in all conditions (autumn 2014: 67°NMF: group mean orientation = 235° ± 41°, *r* = 0.55, *p* < 0.05, *N* = 11, Fig. 3a; 5°NMF: group mean orientation = 204° ± 37°, *r* = 0.57, *p* < 0.05, *N* = 10, Fig. 3b; 5°CMF: group mean orientation = 78° ± 29°, *r* = 0.73, *p* < 0.01, *N* = 9, Fig. 3c). The Mardia-Watson-Wheeler test showed significant differences in the orientation directions between 5°NMF and 5 °CMF (*W* = 12.348, *df* = 2, *p* < 0.002), but not between the 67°NMF and the 5°NMF condition (*W* = 0.574, *df* = 2, *p* > 0.607 (ns)).

In spring 2015, birds were tested in the control conditions (67°NMF and 67 °CMF) and in the 0° inclination conditions (0°NMF and 0°CMF) to investigate further, whether the blackcaps were bimodal or randomly oriented in the field with a flat inclination. The birds oriented significantly in their appropriate migratory direction when they were tested in a magnetic field pointing towards magnetic north with a magnetic inclination of 67° (spring 2015 67°NMF: group mean orientation = 38° ± 28°, *r* = 0.66, *p* < 0.01, *N* = 13, Fig. 4a). When the horizontal component of the 67° inclined field was rotated −120°, the birds turned their orientation accordingly (spring 2015 67°CMF: group mean orientation = 284 ± 22°, *r* = 0.79, *p* < 0.001, *N* = 13, Fig. 4c). The Mardia-Watson-Wheeler test showed a significant difference in the orientation direction between the 67°NMF and the 67°CMF conditions (*W* = 19.965, *df* = 2, *p* < 0.001).

When the birds were tested in the 0° inclination fields (0°NMF and 0°CMF), the birds could not use their magnetic compass anymore (spring 2015 0°NMF: group mean orientation = 8°, *r* = 0.2, *p* = 0.709 (ns), *N* = 9 Fig. 4b; 0°CMF: group mean orientation = 143°, *r* = 0.08, *p* > 0.9 (ns), *N* = 12, Fig. 4d). Furthermore, the orientation at 0° inclination revealed no bimodality of the orientation in the 0°NMF condition (doubled angles: group mean vector = 23°/203°, *r* = 0.11, *p* > 0.9 (ns), *N* = 9), or in the 0°CMF condition (group mean vector = 157°/337°, *r* = 0.32, *p* = 0.299 (ns), *N* = 12). The same holds when the data from the spring seasons 2014–2015 are pooled (pooled data 0°NMF: group mean orientation = 25°, *r* = 0.33, *p* = 0.140 (ns), *N* = 17, Fig. 5a; 0°CMF: group mean orientation = 4°, *r* = 0.08, *p* = 0.882 (ns), *N* = 20, Fig. 5b; double angles: 0°NMF: group mean vector = 115°/295°, *r* = 0.23, *p* = 0.446 (ns), *N* = 17; 0°CMF: group mean vector = 128°/308°, *r* = 0.20, *p* = 0.455 (ns), *N* = 20). If all 0°NMF and 0°CMF data from both seasons are corrected for magnetic north and pooled, also no bimodality was observed (double angles: relative magnetic North: group mean vector = 94°/274°, *r* = 0.20, *p* = 0.513 (ns), *N* = 17).

## Discussion

**Figure 1:**
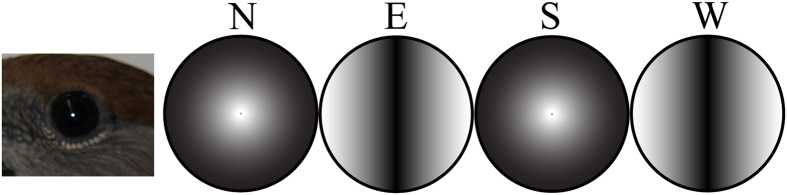
An illustration indicating how the Earth’s magnetic field at the magnetic equator may appear to a bird, which “sees” the magnetic field. Hypothetical signal modulation patterns using the assumptions also used in Ritz *et al*.^26^ and Solov’yov *et al*.^32^ for a bird changing its viewing direction clockwise in 90° increments in a magnetic field of 0° inclination. The four circles represent a full 360° sweep, showing all cardinal directions, from north (left circle) to west (right circle). Each “view” covers 180°.

**Figure 2:**
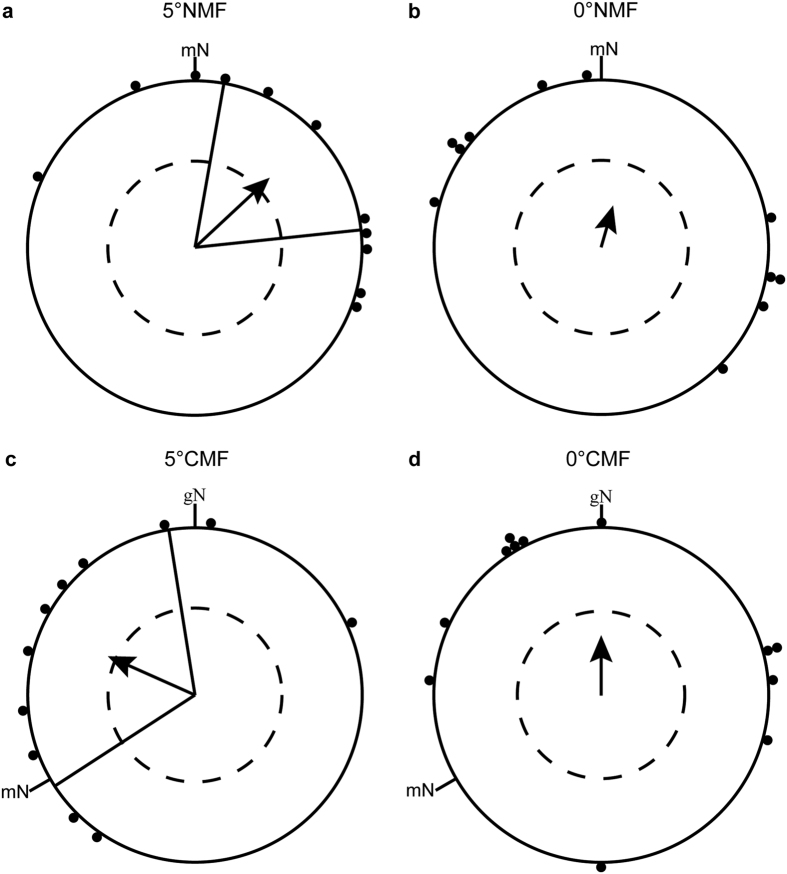
Blackcaps can orient at 5° inclination but become random at 0° inclination. The orientation of the same group of birds in (**a**) an Earth strength magnetic field pointing towards geomagnetic North with an inclination of 5°, (**b**) an Earth strength magnetic field pointing towards geomagnetic North with an inclination of 0°, (**c**) an Earth strength magnetic field pointing towards −120° with an inclination of 5°, and (**d**) an Earth strength magnetic field pointing towards −120° with an inclination of 0°. The magnetic field intensity remained constant in all four conditions and all experiments were done in spring 2014. Each dot at the circle periphery represents the mean orientation angle of an individual bird. The arrows show the group mean directions and vector lengths, the dashed circles indicate the lengths of the group mean vectors needed for significance at the 0.05, 0.01, and 0.001 levels according to the Rayleigh-test, and the straight lines next to the group mean vectors show the 95% confidence interval limits for the group mean headings. mN = magnetic North; gN = geographic North.

**Figure 3:**
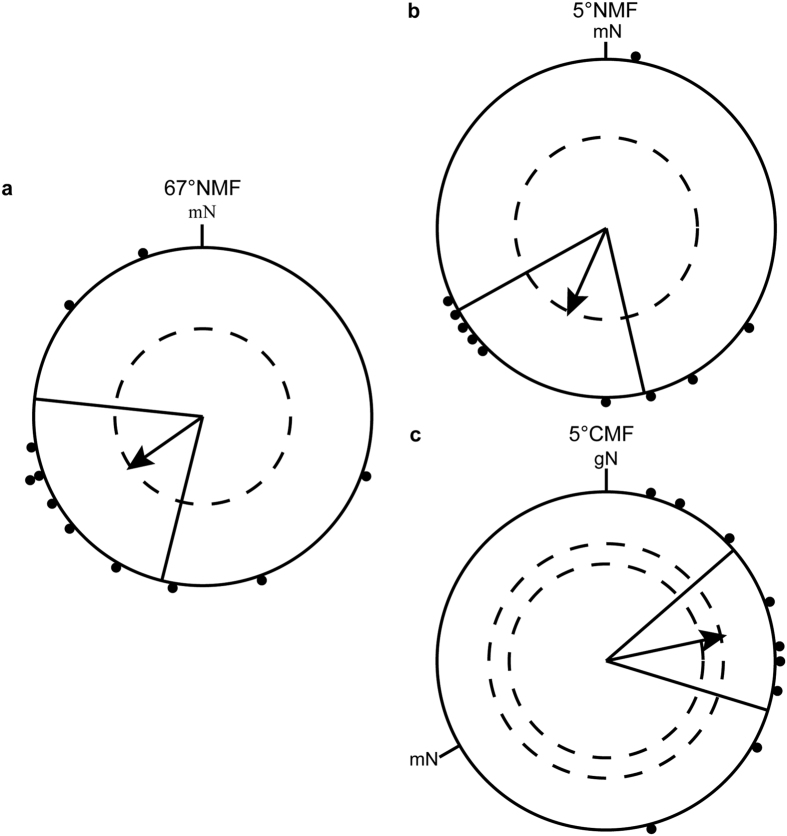
Blackcaps are well oriented at 5° inclination in autumn. (**a**) Unchanged geomagnetic field condition (magnetic inclination angle 67° of Oldenburg); (**b**) inclination set to 5° (5°NMF); (**c**) inclination 5°, horizontal component −120° (5°CMF). For a description of the circular diagrams, see legend to Fig. 2.

**Figure 4:**
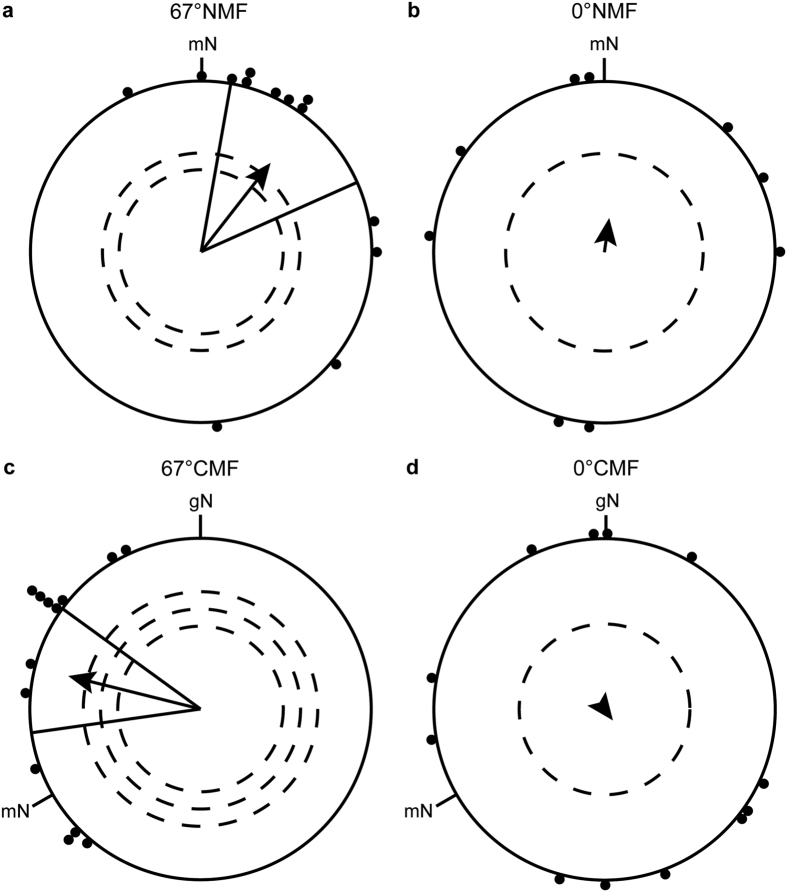
Blackcaps fail to orient in a 0° inclination magnetic field. In the spring migratory season 2015, the blackcaps were well oriented in their natural spring migratory direction in the geomagnetic field of Oldenburg (**a**) and turned their orientation accordingly when the magnetic field was turned 120° counter-clockwise (**c**). In contrast, the birds were randomly oriented when the inclination angle was set to 0° (0°NMF (**b**) and 0°CMF (**d**). For a description of the circular diagrams, see legend to Fig. 2.

**Figure 5:**
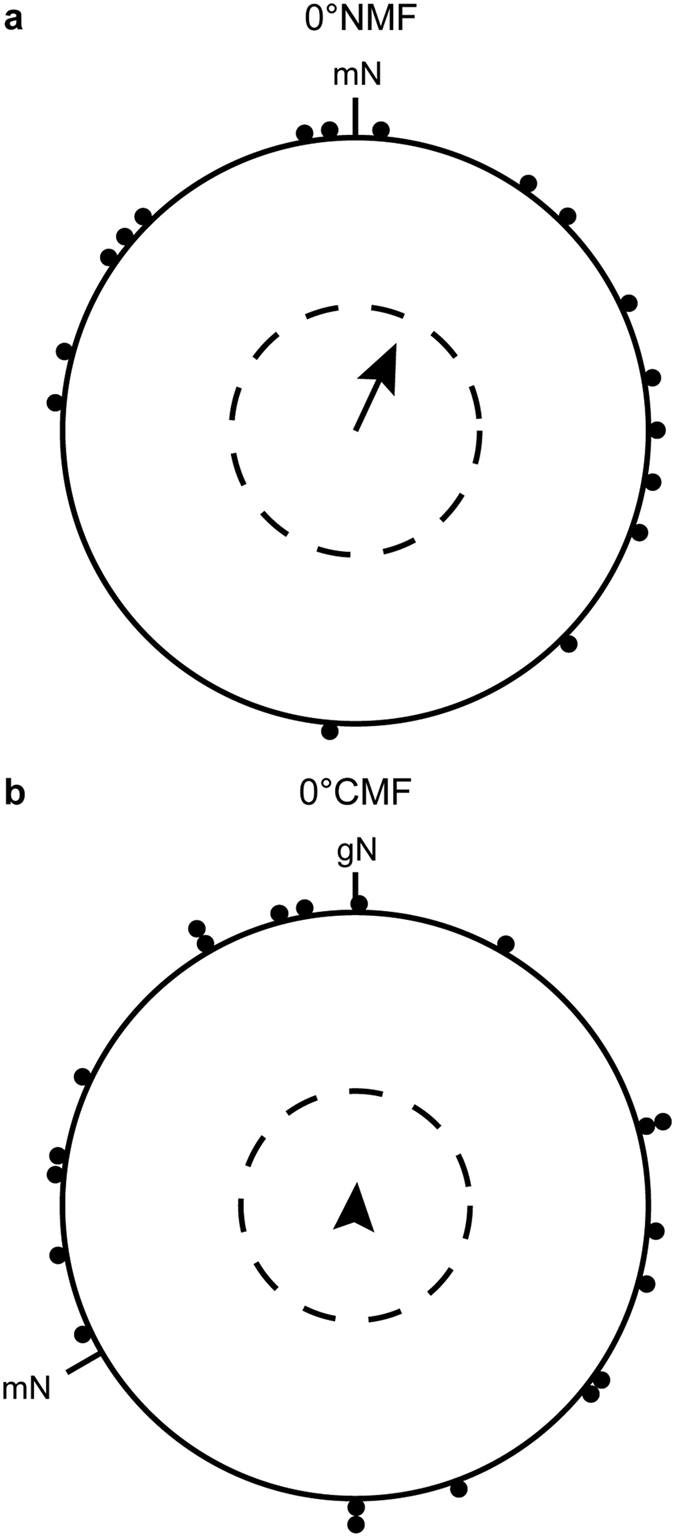
Pooled data from the 0° inclination conditions of spring 2014 and 2015. When the data obtained in the 0° inclination conditions from spring 2014 and spring 2015 are pooled and recalculated for each individual bird, so that each bird only contributes one mean direction seen over the two years in total, it becomes clear that we see no signs of any bimodal NE-SW orientation in the 0°NMF condition or of any E-W bimodal orientation in the 0°CMF condition as might have been expected, if the birds could determine the axis of the magnetic field. (**a**) Pooled data for 0°NMF and (**b**) pooled data for 0°CMF. For a description of the circular diagrams, see legend to Fig. 2.

We could independently confirm the results of previous studies in European robins, *Erithacus rubecula*^9^, and garden warblers, *Sylvia borin*^47^: blackcaps completely fail to orient at 0° inclination, and we saw no robust signs of bimodal orientation. This is surprising because their magnetosensory system should have been able to separate the north-south axis from the east-west axis (see Fig. 1). The fact that they do not show this presumed capability in their orientation behaviour in an Emlen funnel can have many reasons.

One possibility is that the birds’ brains not only consider the absolute sensory information from various potentially navigation relevant cues, but that they also evaluate the quality of the sensory information arriving from each sense^49^. This could take place in one or more candidate brain regions outlined in Mouritsen *et al*.^49^. In that case, the north-south ambiguity, even though it might be in principle detectable, may lead the birds to ignore the magnetic cues altogether.

Another open question is how precisely birds are able to detect magnetic declination angles, and at which minimum inclination angle, the birds are still able to use their magnetic inclination compass. For vertical inclination angles we know that blackcaps are able to use a field with 85° inclination, but not one with 88° inclination^50^. Savannah sparrows tested near the actual magnetic north pole even seemed to be able to orient in a field with 88.6° inclination^51^. In the present study, we could show that blackcaps can orient as well in a magnetic field with a shallow 5° inclination as they can in the normal geomagnetic field found around Oldenburg (67° inclination). Thus, for shallow inclination angles, the limit is certainly also better than 5° as it is for steep inclination angles. Because the Earth’s magnetic field changes ca. 0.009° per km, our results determines the upper limit for the extent of the magnetic compass blind zone around the equator to (2 × 5°)/(0.009°/km) = 1110 km. If the inclination angle detection limit would turn out to be ca. 2–3° as seems to be the case for the steep inclinations, the magnetic compass blind zone would be ca. 440–660 km wide.

A few studies have suggested that exposure to 0° magnetic inclination can trigger a reversal of birds’ orientation direction from “equatorward” to “poleward” and vice versa^44^,^46^,^52^ but see ref. 45. We avoided to expose our birds to 0° inclination in autumn for this reason and to prevent that this field could function as a migratory stop signal in blackcaps, which do not cross the magnetic equator. Because our birds were exposed to the various magnetic inclination conditions in a semi-random pattern, we can state that the blackcaps in our study, which had no stellar cues available, did not reverse their orientation in the control condition, after being exposed to 0° inclination for 1–2 hours several times during spring. Had such exposures to 0° inclination led to orientation direction reversal in spring, the birds tested in our control condition should have become random or bimodal, because the control tests and 0° inclination tests were intermixed throughout spring. However, in the studies observing reorientation^44^,^46^,^52^, the birds were tested in autumn and they were exposed to the 0° inclination for two full days and nights.

In conclusion, blackcaps seem unable to orient in a completely horizontal magnetic field, and their angular determination capabilities related to shallow inclination angles is better than 5°. Considering our current knowledge about the sensitivity of the birds’ magnetic inclination compass, at least two important questions remain: (1) What orientation mechanism(s) do birds use to successfully cross the less than 1110 km broad area around the magnetic equator, where the birds seem unable to use their magnetic inclination compass? Celestial cues would be the most likely solution^2^,^3^. (2) How and at what point are these alternative cues triggered? The point at which the magnetic inclination becomes too shallow to resolve for the birds’ magnetic inclination compass would be one option.

## Materials and Methods

### Test animals

A total of 26 blackcaps (*Sylvia atricapilla*) were tested in this study. All birds were wild-caught in August 2013 and August 2014 within 1 km from the University of Oldenburg, Germany. The birds were housed indoors, two by two, in cages placed in a windowless room with a light regime matching the local photoperiod. The experiments were performed during the migratory seasons in spring and autumn 2014 and during spring 2015 on the Wechloy Campus of the University of Oldenburg. All procedures were performed in accordance with local and national guidelines for the use of animals in research and were approved by the Animal Care and Use Committees of LAVES (Niedersächsisches Landesamt für Verbraucherschutz und Lebensmittelsicherheit, Oldenburg, Germany; protocol log numbers: 33.12-42502-4-07/1422 and 33.12-42502-04-13/1065).

### Magnetic fields

A double-wound, three-axial, Merritt four-coil system (2 × 2 × 2 m) was used to create the experimental magnetic fields^11^,^28^,^48^. The coils were run by three constant current power supplies (KEPCO BOP 50-4M, Kepco Inc., Flushing, NY, USA), one for each axis. The experiments were performed within the center of the coils, where the homogeneity of the field applied by the coils was 99% or better.

First, we pre-tested the blackcaps to make sure they were in migratory mood. Pre-testing was performed in two different field conditions: in the natural geomagnetic field (67°NMF) of Oldenburg (field strength = 48,600 ± 240 nT [standard deviation], inclination = 67.3° ± 0.4°; horizontal direction 360° ± 0.1°), and in a changed magnetic field, in which magnetic North was turned 120° counter clockwise (67°CMF: field strength = 48,600 ± 250 nT; inclination = 67.4° ± 0.3°; horizontal direction = −120° ± 2°). In the NMF condition, the same amount of current that was needed to create the CMF condition was sent through the two subsets of coil windings but in opposite (antiparallel) directions, so that the effective changes in the natural geomagnetic field strength were less than 10 nT.

During the critical experiments, we re-tested the birds under the control conditions (67°NMF and 67°CMF with 67° inclination), and in conditions where the inclination was changed to 5° (5°NMF and 5°CMF performed in spring 2014 and autumn 2014) or 0° (0°NMF and 0°CMF performed in spring 2014 and spring 2015) while the total field intensity remained the same as in the control conditions (Table 1). The magnetic field conditions present inside the funnels were measured daily before the experiments started and remained the same for two consecutive nights per bird.

All magnetic field conditions were set and controlled (Table 1) by a custom-written computer script (MATLAB and Data Acquisition Toolbox™ R2015b, The MathWorks, Inc., Natick, MA, USA) using an analog output module (NI 9263 ± 10 V analog output, 100 kS/s, 4 CH Module, National Instruments, Austin, TX, USA). Furthermore, the magnetic fields with changed inclination was monitored over the whole experimental period by a vector fluxgate magnetometer (FVM-400, Meda, Inc., Dulles, VA, USA) placed under the central funnel and recorded by a custom programmed script (MATLAB and Data Acquisition Toolbox™ R2015b, The MathWorks, Inc., Natick, MA, USA) to confirm the consistency of the magnetic field conditions during the entire experimental period. Due to the inevitable heterogeneities created by the coils, minor deviations from the desired fields could not be avoided, but these were measured and are reported in Table 1.

In spring 2014, the vector fluxgate magnetometer (FVM-400, Meda, Inc., Dulles, VA, USA) was positioned 50 cm under the center of the desk on which the experiments took place. From autumn 2014 onwards, a construction was built, which enabled us to place the vector fluxgate magnetometer (FVM-400, Meda, Inc., Dulles, VA, USA) in exactly the same position every time directly (~4 cm) under the central funnel (at the center of the desk), which improved the consistency and accuracy of the measurements even further.

### Behavioural tests

In spring 2014, the group of tested birds was unusually well oriented^50^. Thus, fewer tests per condition were needed until the group of birds showed a significant orientation in the control condition compared to most other years (compare data given in ref. 50 with data obtained from the same huts in refs 28,29,33,34 and 53).

All behavioural tests took place in wooden huts covered with aluminum plates on the inside, which were grounded and therefore acted as Faraday cages that shielded the inside from time-dependent electromagnetic fields in the frequency range up to ~20 MHz by approximately two orders of magnitude^54^. All electrical equipment (power supplies, computers etc.) was placed outside the experimental room in aluminum-shielded shelves to prevent electromagnetic interferences caused by the equipment to affect the birds. During testing, the room was illuminated with dim, diffused light (2.5 ± 0.25 mW/m^2^) produced by light bulbs (see spectrum given in ref. 28). Hence, the static magnetic field was the only available cue for orientation.

All behavioural tests were conducted in the following way: one hour before the start of the experiments, the birds were placed outdoors in wooden transport boxes fitted with 7 cm diameter mesh-covered peepholes to enable them to see twilight cues and parts of the evening sky. This gives them the possibility to calibrate their magnetic compass^2^,^3^,^55^. At sunset (±10 min), the birds were placed in modified aluminum ‘Emlen funnels’ (35 cm diameter, 15 cm high, walls 45° inclined^56^). The walls of the funnels were lined with scratch-sensitive paper (Blumberg GmbH, Ratingen, Germany) on which the birds’ migratory restlessness became visible as scratches^57^.

Nine birds were tested simultaneously twice each night. The second testing started approximately 1.5 h (±10 min) after the first test started. In the second test round, the birds were switched to another hut or a different funnel position so that we could exclude that the birds had transferred any possible non-magnetic cues from one test to the next.

### Orientation data analyses

After the end of the experiments, all scratch-sensitive papers were evaluated independently by two researchers relative to the overlap point to estimate the mean direction of the scratches. The evaluators did not know in which cardinal direction (N, S, E or W) the overlap point had been fixed. The cardinal direction of the overlap point was chosen randomly and varied in-between test rounds and nights. If the scratch-sensitive paper fulfilled one of the following three criteria it was excluded from further analysis: (1) If the two independently estimated mean directions differed by more than 30°, a third observer was consulted. In the case that no agreement between the three was achievable, the paper was regarded as random (10% of all papers). (2) Papers with less than 30 scratches were classified as inactive, because blackcaps typically left fewer than 30 escape scratches when removed immediately after placing them in Emlen funnels^9^,^29^,^50^ (25% of all papers) (3) If the distribution of the scratches were bimodal (0.3% of all papers). In all other instances, subsequently, the bird’s mean direction was determined as the mean direction of the scratches corrected for the direction of the overlap point. As a result, the number of active and directed tests per bird in the different conditions differed slightly. The average mean heading of an individual bird in a given experimental condition was calculated by addition of unit vectors in each of the mean directions of the individual tests. The group mean vectors were calculated by vector addition of these individual mean directions followed by division by the number of birds tested in the given condition. The significance of the group mean vector was tested using the Rayleigh-test^58^. Differences in group mean orientations between birds tested in different magnetic field conditions were tested by the Mardia-Watson-Wheeler test (MWW; see ref. 58). To test for bimodal orientation, each angle was doubled and the group mean vector of the doubled angles was tested for significance with Rayleigh-test^58^.

## Additional Information

**How to cite this article**: Schwarze, S. *et al*. Migratory blackcaps can use their magnetic compass at 5 degrees inclination, but are completely random at 0 degrees inclination. *Sci. Rep.* **6**, 33805; doi: 10.1038/srep33805 (2016).

## Figures and Tables

**Table 1:** Variability in the experimental magnetic fields used in the present study.

		Magnetic inclination (deg)	Magnetic declination (horizontal polarity) (deg)	Magnetic field flux density (intensity) (nT)
Autumn	67°NMF	68.59 ± 0.06	0 ± 0.14	49,221 ± 103
	5°NMF	5.00 ± 0.03	0 ± 0.27	49,193 ± 121
	5°CMF	5.07 ± 0.04	−120 ± 0.21	49,079 ± 79
Spring	67°NMF	68.21 ± 0.15	0 ± 1.91	49,543 ± 217
	67°CMF	68.13 ± 0.18	−120 ± 1.91	49,276 ± 245
	0°NMF	0.05 ± 0.05	0 ± 2.03	49,543 ± 237
	0°CMF	0.05 ± 0.06	−120 ± 1.96	49,357 ± 334

Data are means ± s.d. measured in the center of the coil system; a magnetometer probe was positioned directly under the experimental table in the middle of the coil system, enabling a continuous recording of the magnetic field components during the experiments.

## References

[b1] Able K. P. & Able M. A. Development of sunset orientation in a migratory bird: no calibration by the magnetic field. Anim. Behav. 53, 363–368 (1997).

[b2] Cochran W. W., Mouritsen H. & Wikelski M. Migrating songbirds recalibrate their magnetic compass daily from twilight cues. Science 304, 405–408 (2004). 15087541 10.1126/science.1095844

[b3] Muheim R., Moore F. R. & Philips B. Calibration of magnetic and celestial compass cues in migratory birds - a review of cue-conflict experiments. J. Exp. Biol. 209, 2–17 (2006). 16354773 10.1242/jeb.01960

[b4] Sauer F. Die Sternenorientierung nächtlich ziehender Grasmücken (*Sylvia atricapilla, borin und curruca*). Z. Tierpsychol. 14, 29–70 (1957).

[b5] Emlen S. T. The stellar-orientation system of a migratory bird. Sci. Am. 233, 102–111 (1975). 1145171 10.1038/scientificamerican0875-102

[b6] Mouritsen H. & Larsen O. N. Migrating songbirds tested in computer-controlled Emlen funnels use stellar cues for a time-independent compass. J. Exp. Biol. 204, 3855–3865 (2001). 11807103 10.1242/jeb.204.22.3855

[b7] Michalik A., Alert B., Engels S., Lefeldt N. & Mouritsen H. Star compass learning: how long does it take? J. Ornithol. 155, 225–234 (2014).

[b8] Alert B., Michalik A., Helduser S., Mouritsen H. & Güntürkün O. Perceptual strategies of pigeons to detect a rotational centre - a hint for star compass learning? PLoS One 10, e0119919 (2015). 25807499 10.1371/journal.pone.0119919 PMC4373800

[b9] Wiltschko W. & Wiltschko R. Magnetic compass of European robins. Science 176, 62–64 (1972). 17784420 10.1126/science.176.4030.62

[b10] Wiltschko R. & Wiltschko W. Magnetic Orientation in Animals. (Berlin, Springer-Verlag, 1995).

[b11] Mouritsen H. The magnetic xsenses. In Neurosciences - From Molecule to Behavior: a university textbook (ed. Galizia C. G. & Lledo P.-M.) 427–443 (Berlin Springer Verlag, 2013).

[b12] Mouritsen H. Magnetoreception in birds and its use for long-distance migration. In Sturkie’s Avian Physiology (ed. Scanes C.) 113–133 (Academic Press, 2015).

[b13] Winklhofer M., Holtkamp-Rötzler E., Hanzlik M., Fleissner G. & Petersen N. Clusters of superparamagnetic magnetite particles in the upper-beak skin of homing pigeons: evidence of a magnetoreceptor? Eur. J. Mineral. 13, 659–669 (2001).

[b14] Mora C. V., Davison M., Martin Wild J. & Walker M. M. Magnetoreception and its trigeminal mediation in the homing pigeon. Nature 432, 508–511 (2004). 15565156 10.1038/nature03077

[b15] Heyers D., Zapka M., Hoffmeister M., Wild J. M. & Mouritsen H. Magnetic field changes activate the trigeminal brainstem complex in a migratory bird. Proc. Natl. Acad. Sci. USA 107, 9394–9399 (2010). 20439705 10.1073/pnas.0907068107 PMC2889125

[b16] Kishkinev D., Chernetsov N., Heyers D. & Mouritsen H. Migratory reed warblers need intact trigeminal nerves to correct for a 1,000 km eastward displacement. PLoS One 8, e65847 (2013). 23840374 10.1371/journal.pone.0065847 PMC3694148

[b17] Lefeldt N. . Magnetic field-driven induction of ZENK in the trigeminal system of pigeons (*Columba livia*). J. R. Soc. Interface 11, 20140777 (2014). 25232052 10.1098/rsif.2014.0777 PMC4191110

[b18] Kishkinev D. Sensory mechanisms of long-distance navigation in birds: a recent advance in the context of previous studies. J. Ornithol. 156, 145–161 (2015).

[b19] Kishkinev D., Chernetsov N., Pakhomov A., Heyers D. & Mouritsen H. Eurasian reed warblers compensate for virtual magnetic displacement. Curr. Biol. 25, R822–R824 (2015). 26439333 10.1016/j.cub.2015.08.012

[b20] Falkenberg G. . Avian magnetoreception: elaborate iron mineral containing dendrites in the upper beak seem to be a common feature of birds. PLoS One 5, e9231 (2010). 20169083 10.1371/journal.pone.0009231 PMC2821931

[b21] Winklhofer M. & Kirschvink J. L. A quantitative assessment of torque-transducer models for magnetoreception. J. R. Soc. Interface 7, S273–S289 (2010). 20086054 10.1098/rsif.2009.0435.focus PMC2843997

[b22] Mouritsen H. Sensory biology: search for the compass needles. Nature 484, 320–321 (2012). 22517155 10.1038/484320a

[b23] Treiber C. D. . Clusters of iron-rich cells in the upper beak of pigeons are macrophages not magnetosensitive neurons. Nature 484, 367–370 (2012). 22495303 10.1038/nature11046

[b24] Schulten K., Swenberg C. E. & Weller A. A biomagnetic sensory mechanism based on magnetic field modulated coherent electron spin motion. Z Phys. Chem. 111, 1–5 (1978).

[b25] Wiltschko W., Munro U., Ford H. & Wiltschko R. Magnetic inclination compass: a basis for the migratory orientation of birds in the northern and southern hemisphere. Experientia 49, 167–170 (1993).

[b26] Ritz T., Adem S. & Schulten K. A model for photoreceptor-based magnetoreception in birds. Biophys. J. 78, 707–718 (2000). 10653784 10.1016/S0006-3495(00)76629-X PMC1300674

[b27] Wiltschko R. & Wiltschko W. Magnetoreception. BioEssays 28, 157–168 (2006). 16435299 10.1002/bies.20363

[b28] Zapka M. . Visual but not trigeminal mediation of magnetic compass information in a migratory bird. Nature 461, 1274–1277 (2009). 19865170 10.1038/nature08528

[b29] Hein C. M. . Night-migratory garden warblers can orient with their magnetic compass using the left, the right or both eyes. J. R. Soc. Interface 7, S227–S233 (2010). 19889693 10.1098/rsif.2009.0376.focus PMC2844002

[b30] Lau J. C. S., Wagner-Rundell N., Rodgers C. T., Green N. J. B. & Hore P. J. Effects of disorder and motion in a radical pair magnetoreceptor. J. R. Soc. Interface 7, S257–S264 (2010). 20007172 10.1098/rsif.2009.0399.focus PMC2844003

[b31] Ritz T., Ahmad M., Mouritsen H., Wiltschko R. & Wiltschko W. Photoreceptor-based magnetoreception: optimal design of receptor molecules, cells, and neuronal processing. J. R. Soc. Interface 7, S135–S146 (2010). 20129953 10.1098/rsif.2009.0456.focus PMC2843994

[b32] Solov’yov I. A., Mouritsen H. & Schulten K. Acuity of a cryptochrome and vision-based magnetoreception system in birds. Biophys. J. 99, 40–49 (2010). 20655831 10.1016/j.bpj.2010.03.053 PMC2895366

[b33] Hein C. M., Engels S., Kishkinev D. & Mouritsen H. Robins have a magnetic compass in both eyes. Nature 471, E11–E13 (2011). 21455128 10.1038/nature09875

[b34] Engels S., Hein C. M., Lefeldt N., Prior H. & Mouritsen H. Night-migratory songbirds possess a magnetic compass in both eyes. PLoS One 7, e43271 (2012). 22984416 10.1371/journal.pone.0043271 PMC3440406

[b35] Lau J. C. S., Rodgers C. T. & Hore P. J. Compass magnetoreception in birds arising from photo-induced radical pairs in rotationally disordered cryptochromes. J. R. Soc. Interface 9, 3329–3337 (2012). 22977104 10.1098/rsif.2012.0374 PMC3481564

[b36] Solov’yov I. A., Domratcheva T., Shahi A. R. M. & Schulten K. Decrypting cryptochrome: revealing the molecular identity of the photoactivation reaction. J. Am. Chem. Soc. 134, 18046–18052 (2012). 23009093 10.1021/ja3074819 PMC3500783

[b37] Hiscock H. G. . The quantum needle of the avian magnetic compass. Proc. Natl. Acad. Sci. 113, 4634–4639 (2016). 27044102 10.1073/pnas.1600341113 PMC4855607

[b38] Hore P. J. & Mouritsen H. The radical-pair mechanism of magnetoreception. Annu. Rev. Biophys. 45, 299–344 (2016). 27216936 10.1146/annurev-biophys-032116-094545

[b39] Kattnig D. R. . Chemical amplification of magnetic field effects relevant to avian magnetoreception. Nat. Chem. 8, 384–391 (2016). 27001735 10.1038/nchem.2447

[b40] Alerstam T. Bird Migration (Cambridge University Press, 1993).

[b41] Bairlein F. Results of bird ringing in the study of migration routes and behaviour. Ardea 89, 7–19 (2001).

[b42] Bairlein F. . Cross-hemisphere migration of a 25 g songbird. Biol. Lett. 8, 505–507 (2012). 22337504 10.1098/rsbl.2011.1223 PMC3391447

[b43] Schmaljohann H., Buchmann M., Fox J. W. & Bairlein F. Tracking migration routes and the annual cycle of a trans-Sahara songbird migrant. Behav. Ecol. Sociobiol. 66, 915–922 (2012).

[b44] Wiltschko W. & Wiltschko R. Migratory orientation: magnetic compass orientation of garden warblers (*Sylvia borin*) after a simulated crossing of the magnetic equator. Ethology 91, 70–74 (1992).

[b45] Beason R. C. You can get there from here: responses to simulated magnetic equator crossing by the bobolink (*Dolichonyx oryzivorus*). Ethology 91, 75–80 (1992).

[b46] Beason R. C. Use of an inclination compass during migratory orientation by the bobolink (*Dolichonyx oryzivorus*). Ethology 81, 291–299 (1989).

[b47] Wiltschko W. Der Magnetkompass der Gartengrasmücke (*Sylvia borin*). J. Ornithol. 115, 1–7 (1974).

[b48] Kirschvink J. L. Uniform magnetic fields and double-wrapped coil systems: improved techniques for the design of bioelectromagnetic experiments. Bioelectromagnetics 13, 401–411 (1992). 1445421 10.1002/bem.2250130507

[b49] Mouritsen H., Heyers D. & Güntürkün O. The neural basis of long-distance navigation in birds. Annu. Rev. Physiol. 78, 133–154 (2016). 26527184 10.1146/annurev-physiol-021115-105054

[b50] Lefeldt N., Dreyer D., Schneider N.-L., Steenken F. & Mouritsen H. Migratory blackcaps tested in Emlen funnels can orient at 85 degrees but not at 88 degrees magnetic inclination. J. Exp. Biol. 218, 206–211 (2015). 25452505 10.1242/jeb.107235

[b51] Åkesson S., Morin J., Muheim R. & Ottosson U. Avian orientation at steep angles of inclination: experiments with migratory white-crowned sparrows at the magnetic north pole. Proc. R. Soc.Lond., B, Biol. Sci. 268, 1907–1913 (2001). 10.1098/rspb.2001.1736 PMC1088826 11564346

[b52] Beason R. C. Interaction of visual and non-visual cues during migratory orientation by the bobolink (*Dolichonyx oryzivorus*). J. Ornithol. 128, 317–324 (1987).

[b53] Schwarze S. . Weak broadband electromagnetic fields are more disruptive to magnetic compass orientation in a night-migratory songbird (*Erithacus rubecula*) than strong narrow-band fields. Front. Behav. Neurosci. 10, 55 (2016). 27047356 10.3389/fnbeh.2016.00055 PMC4801848

[b54] Engels S. . Anthropogenic electromagnetic noise disrupts magnetic compass orientation in a migratory bird. Nature 509, 353–356 (2014). 24805233 10.1038/nature13290

[b55] Chernetsov N., Kishkinev D., Kosarev V. & Bolshakov C. V. Not all songbirds calibrate their magnetic compass from twilight cues: a telemetry study. J. Exp. Biol. 214, 2540–2543 (2011). 21753048 10.1242/jeb.057729

[b56] Emlen S. T. & Emlen J. T. A technique for recording migratory orientation of captive birds. Auk 83, 361–367 (1966).

[b57] Mouritsen H., Feenders G., Hegemann A. & Liedvogel M. Thermal paper can replace typewriter correction paper in Emlen funnels. J. Ornithol. 150, 713–715 (2009).

[b58] Batschelet E. Circular Statistics in Biology. (Academic Press, 1981).

